# Comparison of 1-cm Versus 2-cm Excision Margins for Cutaneous Melanoma: A Systematic Review and Meta-Analysis

**DOI:** 10.7759/cureus.84848

**Published:** 2025-05-26

**Authors:** Darya Pospyelova, Isabel Bunola-Hadfield, Martha F De La Cruz Monroy, Mahmoud A Kandil, Lauren-Jane Fredericks-Bowyer, Haris Duvnjak, Himani Murdeshwar, Shafiq Rahman

**Affiliations:** 1 General Surgery, Leeds Teaching Hospitals NHS Trust, Leeds, GBR; 2 Plastic and Reconstructive Surgery, York and Scarborough Teaching Hospitals NHS Foundation Trust, Scarborough, GBR; 3 Plastic and Reconstructive Surgery, Bradford Teaching Hospitals NHS Trust, Bradford, GBR; 4 Plastic and Reconstructive Surgery, University Hospitals of North Durham, Durham, GBR; 5 Plastic and Reconstructive Surgery, Nottingham University Hospitals NHS Trust, Nottingham, GBR; 6 Plastic and Reconstructive Surgery, Northern Care Alliance NHS Foundation Trust, Salford, GBR; 7 Plastic and Reconstructive Surgery, Barts Health NHS Trust, London, GBR; 8 Plastic and Reconstructive Surgery, Hull Teaching Hospitals NHS Trust, Hull, GBR

**Keywords:** 5-year disease free survival, breslow thickness, cutaneous malignant melanoma, melanoma surgery, research in melanoma

## Abstract

Surgical intervention is the primary treatment for localised cutaneous melanoma, with wide local excision being the gold standard. However, optimal excision margins remain a point of debate amongst clinicians. This systematic review and meta-analysis evaluated the outcomes of 1 cm versus 2 cm excision margins for intermediate to thick melanomas.

Following the Preferred Reporting Items for Systematic Reviews and Meta-Analyses (PRISMA) guidelines, a search of databases including PubMed, Medline, Cumulative Index to Nursing and Allied Health Literature (CINAHL) and the Cochrane Central Register of Controlled Trials (CENTRAL) was conducted. The World Health Organization International Clinical Trials Registry, ClinicalTrials.gov, the ISRCTN registry and Science Direct were also screened. Seven studies met the inclusion criteria. Primary outcomes included overall, locoregional, and distant melanoma recurrence rates between 1 cm and 2 cm margins. Secondary outcomes assessed five-year disease-free survival and reconstruction complexity. The results were presented with forest plots at 95% confidence interval.

No significant difference in overall recurrence rate was observed between 1 cm and 2 cm margins (0.846; 0.649, 1.103, p=0.216). Three studies reported no significant difference in disease-free survival at final follow-up. Primary closure was more achievable with a 1 cm margin (1.300; 1.053, 1.605, p value <0.015), while 2 cm margins required more complex reconstructions such as local flaps or skin grafts (0.772; 0.625, 0.955, p< 0.017).

This systematic review and meta-analysis suggests that a 1 cm margin is as safe as a 2 cm margin for intermediate to thick melanomas, reducing the need for complex reconstructions. Further randomised controlled trials are recommended to solidify these findings, but this study provides a strong foundation for adopting smaller excision margins.

## Introduction and background

Melanoma is the third most prevalent skin cancer in the UK [1]. In 2022, it accounted for 4% of all new cancer diagnoses [1,2]. Between 2014 and 2035, the incidence of melanoma in the UK is projected to increase by 7%, reaching 32 cases per 100,000 [2]. Surgical intervention remains the primary approach for localised cutaneous melanoma, with wide local excision of the biopsy site being the gold standard. 

The Breslow thickness of the tumour determines the recommended excision margins for melanoma. However, there is some inconsistency in the literature and among clinicians regarding these excision margins. In 2022, the updated guidelines from the National Institute for Health and Care Excellence (NICE) recommend a 1 cm margin for stage I melanomas, which include tumours with a thickness of less than 1 mm or 1-2 mm without ulceration. For stage II melanomas, which consist of tumours that are 1-2 mm with ulceration or greater than 2 mm, a 2 cm margin is advised [3]. In contrast, the National Comprehensive Cancer Network (NCCN) advises a 1 cm margin for tumours less than 1 mm thick, a 1-2 cm margin for tumours 1-2 mm thick, and a 2 cm margin for tumours 2-4 mm thick [4]. An adequate wide local excision margin reduces the likelihood of leaving behind microscopic disease. However, the recommended margins can be adjusted based on individual patient factors such as anatomical location or functional concerns, making the surgical management of melanoma an area of ongoing debate [4,5]. Therefore, establishing appropriate margins is critical to improving survival and quality of life for patients with melanoma.

Several studies have investigated the optimal excision margins for intermediate-risk melanomas (lesions with thicknesses between 1.01 and 4.0 mm) and high-risk melanomas (lesions thicker than 4.0 mm), but findings have been conflicting [6]. A randomised controlled trial by Thomas et al. [7] in 2004 indicated that patients with high-risk melanomas had a higher risk of locoregional recurrence when treated with a 1 cm margin compared to a 3 cm margin. However, other researchers, including Hunger et al. [5] and Hudson et al. [8], found no significant difference in recurrence rates between patients with intermediate or high-risk melanomas who underwent excision with 1 cm versus 2 cm margins. Additionally, Koskiuvo et al. [9] evaluated melanomas <2 mm and reported no recurrences with 1 cm margins.

The data shows variability when evaluating the overall survival rates or melanoma-specific survival [10]. Thomas et al. [7] found no significant difference in either overall or melanoma-specific survival. Hudson et al. [8] and Hunger et al. [5] support this finding. However, Doepker et al. [11] observed that the survival rates were lower in the 1 cm excision margin group for melanomas measuring less than 2 mm despite having similar recurrence rates. Furthermore, Hayes et al. [12] linked 1 cm margins to a higher melanoma-specific mortality rate than 3 cm margins for melanomas greater than 2 mm, suggesting that wider margins may benefit high-risk cases. A review from 2016 indicated that margins of 1-2 cm were associated with poorer survival compared to margins of 3-5 cm [13]. Conversely, a meta-analysis from 2002 found no differences in recurrence or mortality between narrow (1-2 cm) and wide (3-5 cm) margins [14]. However, no meta-analysis has specifically compared 1 cm and 2 cm margins for intermediate (1.01 to 4.0 mm) to thick (>4.0 mm) melanomas.

This systematic review and meta-analysis aims to evaluate treatment outcomes and closure techniques in intermediate to thick cutaneous melanomas comparing 1 cm versus 2 cm excision margins.

The findings of this study were previously presented as a poster presentation at the 49th Association of Surgeons in Training Conference (ASiT) Annual Surgical Conference on 9th March, 2025.

## Review

Materials and methods

This systematic review and meta-analysis followed the Preferred Reporting Items for Systematic Reviews and Meta-Analysis statement standards (PRISMA) [15].

Eligibility Criteria

All comparative studies, including randomised controlled trials, non-randomised controlled trials, observational studies, and case-control studies comparing 1 cm versus 2 cm excision margins for cutaneous melanomas, were included. There was no restriction on age, co-morbidity status, sex or melanoma subtype; however, thin melanomas with a Breslow thickness of less than 1 mm were excluded. Case reports, review articles, book chapters, editorials, short communications and correspondences were all excluded, as well as those not reported in English. In addition, studies that were not relevant to comparing 1 cm versus 2 cm excision margins were also excluded from the review.

Outcome Measures

The primary outcome measures included overall, locoregional, and distant melanoma recurrence rates. Secondary outcomes included five-year disease-free survival and the complexity of reconstruction necessitated in both 1 cm and 2 cm excision cohorts.

Literature Identification

An extensive search of the following electronic databases was conducted: PubMed, Medline, Cumulative Index to Nursing and Allied Health Literature (CINAHL), and the Cochrane Central Register of Controlled Trials (CENTRAL). In addition, the World Health Organization International Clinical Trials Registry, ClinicalTrials.gov, the ISRCTN registry and Science Direct were also screened. The last search was conducted on 21st September 2024. The search terms consisted of “1cm”, “2cm”, “excision margins”, “wide local excision”, “cutaneous or skin melanoma”, “intermediate thickness” and “thick melanomas”.

Study Screening and Eligibility

Two authors, SR and IBH, independently assessed the titles and abstracts of the articles retrieved from the literature search. Articles that met the eligibility criteria were selected, and their full texts were reviewed. 

Data Extraction and Management

The collected data included the study characteristics and patient demographics, Breslow thickness, length of follow-up, the number of patients with overall, locoregional, and distant recurrences, five-year disease-free survival rates, the number of patients undergoing primary closure, and those receiving skin grafts or flaps. Five authors independently extracted and recorded the data, with any ambiguities resolved through discussions.

Data Synthesis

Data analysis was performed using OpenMetaAnalyst software (Created by Brown University), applying a random effects model. Forest plots were generated with 95% confidence intervals. For dichotomous outcome data, odds ratios were calculated. To mitigate for variations of heterogeneity in the data, a generic inverse variance function was adopted for all analyses.

Methodological Quality and Risk of Bias

The quality of all non-randomised studies was assessed using the Newcastle-Ottawa Scale (NOS), which employs a star rating system [16]. This evaluates studies based on three key criteria: the selection of study groups, the comparability of these groups, and the ascertainment of the exposure or outcome of interest. Each study can be awarded up to a maximum of nine stars [16]. The Cochrane Risk of Bias Assessment tool was used to assess the quality of the randomised-controlled study [17].

Assessment of Heterogeneity

The heterogeneity of all included studies was evaluated using the Cochran Q test and the I2 score. Heterogeneity in relation to I2 was interpreted on the following scale: 0-25% was regarded low, 25-75% moderate, and 75-100% high.

Results

**Figure 1:**
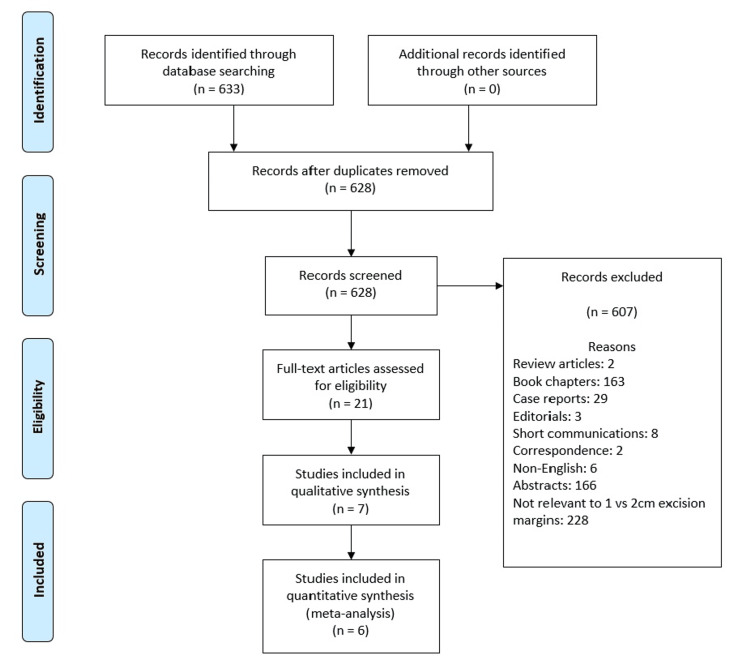
Preferred Reporting Items for Systematic Reviews and Meta-Analyses (PRISMA) flow chart of study selection.

The systematic search of databases resulted in 633 articles, of which seven met the inclusion criteria, as shown in Figure 1 [15]. Table 1 and Table 2 show the results of the primary and secondary outcomes.

**Table 1:** Amalgamation of patient characteristics, study design, average Breslow thickness, follow-up, recurrences, disease-free period as well as closure technique. N/R: Not reported, *Four additional cases in an unclear group (i.e.: either 1 cm or 2 cm).

Study	Origin population	Study design	Patient number & gender	Mean age (years)	Mean Breslow thickness (mm)	Length of follow-up (months)	Overall Recurrence(n)	Locoregional recurrence (n)		Distant recurrence (n)		Disease-free period (n)		Primary closure (n)		Skin graft/flap (n)	
								1cm	2cm	1cm	2cm	1cm	2cm	1cm	2cm	1cm	2cm
Hudson et al, 2013 [8]	Single institution, USA	Retrospective cohort study	576 M= 335 F = 241 1cm = 224 2cm = 352	52.6	1cm: 1.27 2cm: 1.48	1cm: 28 2cm: 43	55	17	20	7	11	NR	NR	181	305	43	45
Koskivuo et al, 2015 [9]	Single institution, Finland	Retrospective cohort study	160 M = 64 F= 96 1cm = 80 2cm = 80	61.6	1cm: 1.7 2cm: 1.8	1cm: 50 2cm: 57	29	6	9	8	6	58	58	62	36	18	44
Haydu et al, 2016 [18]	Melanoma Institute Australia	Retrospective cohort study	2131 M=1257 F = 874 1cm = 326 2cm = 1805	55	1.46	57	265	268		161		5-year cumulative disease-free survival 1cm: 344 2cm: 463		NR		NR	
Rawlani et al, 2015 [19]	Single institution, USA	Retrospective cohort study with prospective follow up	79 M = 46 F = 33 1cm = 37 2cm = 42	1cm = 51.6 2cm = 54.3	<1.0mm: 38 1.01-2mm: 21 2.01-4mm: 12 >4.0mm: 8	1cm: 68.2 2cm: 74.1	18	4*	3	7 (unclear group)		No data on excision margins just a differentiation between recommended and reduced margins		No data on excision margins just a differentiation between recommended and reduced margins		No data on excision margins just a differentiation between recommended and reduced margins	
Moncrieff et al, 2018 [20]	MelMarT trial (UK, USA, Australia, Canada, Sweden)	Randomised Controlled Trial (RCT)	377 M=211 F=186 1cm= 185 2cm = 192	1cm = 58.97 2cm: 58.19	1cm: 2.12 2cm: 2.27	12	NR	NR		NR		NR		159	125	25	67
Hunger et al, 2015 [5]	Single institution, Switzerland	Retrospective cohort study	325 M=187 F = 138 1cm = 228 2cm = 663	61.8	1cm: 4.22 2cm: 4.67	1cm: 62 2cm: 66	162	55	30	52	25	Mean disease free survival estimate in days 1cm: 3289 2cm: 2139		NR		NR	
Doepker et al, 2016 [11]	Single institution, USA	Retrospective cohort study	965 M = 592 F = 373 1cm = 302 2cm = 663	Median 1cm: 67 2cm: 63	Median 1cm: 1.3 2cm: 1.4	60	105	18	46	11	30	NR		208	452	94	211

**Table 2:** Anatomical location of melanomas in included studies.

Study	Anatomical location			
	Head and neck	Trunk	Upper limb	Lower limb
Hudson et al, 2013 [8]	94	233	137	112
Koskivuo et al, 2015 [9]	48	50	32	30
Rawlani et al, 2015 [19]	79	-	-	-
Haydu et al, 2016 [18]	372	855	904	
Hunger et al, 2015 [5]	58	128	60	79
Doepker et al, 2016 [11]	195	330	275	165
Moncrieff et al, 2018 [20]	12	102	70	
Total (n = 4,613)	858 (18.6%)	1,698 (36.8%)	1,864 (42.6%)	

Primary outcomes

Overall Recurrence

**Figure 2:**
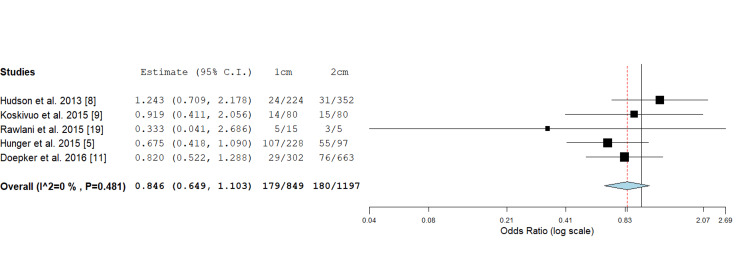
Odds ratio assessment for overall melanoma recurrence: 0.846 (0.649, 1.103), P value = 0.216.

Five studies reported on the overall recurrence rate of cutaneous melanomas comparing 1 cm versus 2 cm wide local excision margins. An insignificant difference was observed in the odds ratio assessment (0.846; 0.649, 1.103, P value = 0.216) for the two cohorts (Figure 2).

Locoregional Recurrence

**Figure 3:**
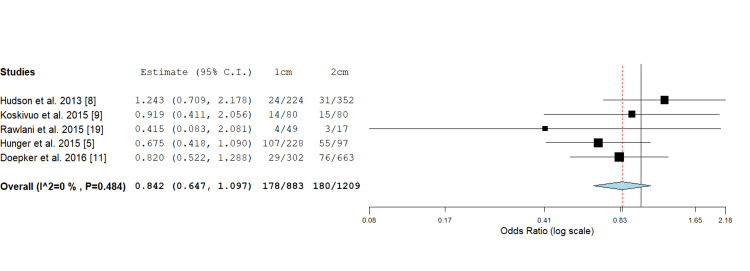
Odds ratio assessment of cutaneous melanoma locoregional recurrence rate: 0.842 (0.647, 1.097), P value = 0.202.

Five studies reported on locoregional recurrence in cutaneous melanoma comparing 1 cm versus 2 cm excision margins. There was no significant difference in the rate of locoregional recurrence on odds ratio assessment (0.842; 0.647, 1.097, P value = 0.202) (Figure 3). 

Distant Recurrence

**Figure 4:**
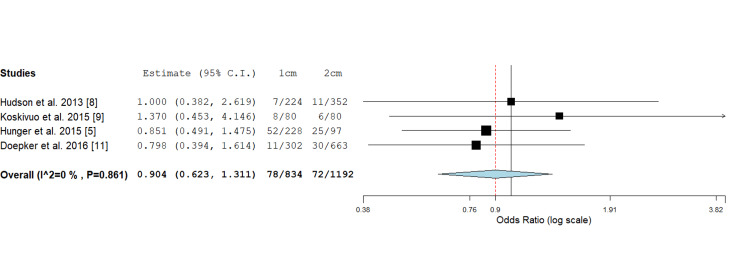
Odds ratio assessment of cutaneous melanoma distant recurrence rate: 0.904 (0.623, 1.311), P value = 0.593.

Four studies reported the distant recurrence rate in cutaneous melanomas by comparing local excision margins of 1 cm versus 2 cm. The odds ratio assessment showed no significant difference between the two margin widths, with values of 0.904; 0.623, and 1.311, and a P value of 0.593 (Figure 4). 

Secondary outcomes

Disease-Free Period

Three studies reported on disease-free survival. Haydu et al. [18] extrapolated clearance margins from pathological specimens and found that the five-year cumulative disease-free survival rates were 80.9% (n=344) for 1 cm margins and 84.5% (n=463) for 2 cm margins, respectively. The difference was not statistically significant, with a p-value of 0.202 [18]. This finding was further supported by a multivariate analysis that also showed no significant difference between the two groups [18]. Koskivuo et al. [9] reported disease-free survival in both the 1 cm and 2 cm excision margins to be 72.5% (n=58) at the final follow-up with a mean follow-up duration of 53 months. Again, no significant difference (p=0.977) was observed using the Kaplan-Meier method. Hunger et al. [5] calculated mean disease-free survival in days to be 3,289 days (approximately nine years) for the 1 cm clearance margin and 2,139 days (approximately 5.86 years) for the 2 cm margin. Both Kaplan-Meier methods and Cox regression analysis indicated no significant difference between the two groups, with Cox regression producing an estimated hazard ratio of 0.948 (95% CI: 0.627-1.433) [5].

Both Hudson et al. [8] and Rawlani et al. [19] reported disease-free survival in a manner that did not allow for comparison between groups based on excision margins.

Primary Closure

**Figure 5:**
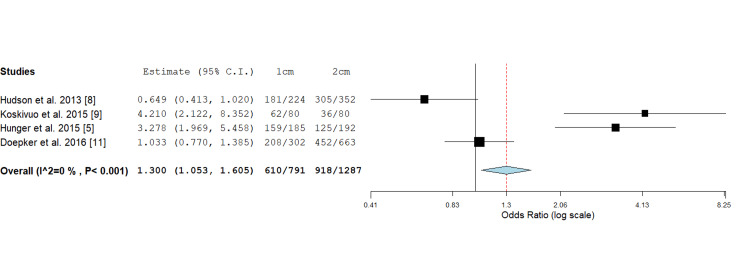
Odds ratio assessment of cutaneous primary closure in 1 cm versus 2 cm wide local rescission margins: 1.300 (1.053, 1.605), P value <0.015.

Four studies reported on primary closure of 1 cm versus 2 cm wide local excision margins. A statistically significant difference was observed using odds ratio assessment, with more primary closures being achieved when a 1 cm margin was taken (1.300; 1.053, 1.605, P value < 0.015) (Figure 5). Koskivuo et al. [9] and Moncrieff et al. [20] both showed a significantly higher rate of primary closure in the 1cm excision group compared to the 2 cm group. However, Hudson et al. [8] and Doepker et al. [11] showed no significant difference between the two groups. 

Skin Graft/Local Flap

**Figure 6:**
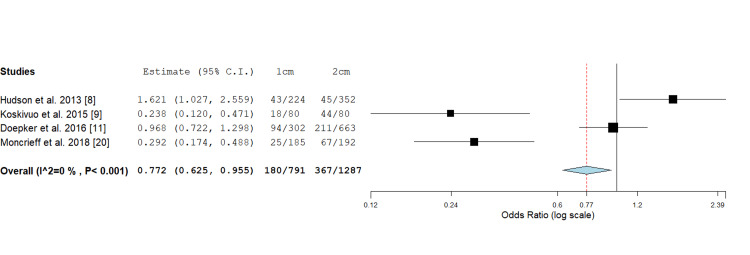
Odds ratio assessment of skin graft/local flap in 1 cm versus 2 cm wide local excision margins: 0.772 (0.625, 0.955), P value <0.017.

Four studies in total reported on the incidence of skin graft/ local flap in 1 cm versus 2 cm wide local excision margin. The 2 cm excision margin group required a significantly higher rate of complex reconstructions, including local flaps or skin graft coverage, compared to the 1 cm group, as observed through odds assessment (0.772; 0.625, 0.955, P value < 0.017) (Figure 6).

Studies' quality and bias

Table 3 shows the assessment of the quality of non-randomised studies while Table 4 shows the Cochrane Risk of Bias assessment.

**Table 3:** Newcastle-Ottawa Scale (NOS) to assess the quality of non-randomised studies.

Study	Selection	Comparability	Outcome
Hudson et al. 2013 [8]	***		*
Koskivuo et al. 2015 [9]	***	*	**
Rawlani et al. 2015 [19]	***	*	***
Hunger et al. 2015 [5]	***	*	*
Haydu et al. 2016 [18]	***		*
Doepker et al. 2016 [11]	***		*

**Table 4:** Cochrane Risk of Bias assessment

Domain in Moncrieff et al. 2018 [20]	Review authors’ judgement	Support for judgement
Selection bias Random sequence generation	Low risk	Patients were randomised electronically in a 1:1 fashion
Selection bias Allocation concealment	Unclear risk	No information given. However, the baseline differences between intervention groups did not suggest a problem with the randomisation process
Reporting bias Selective reporting	Low risk	Data analysed in accordance with pre-defined protocol. Numerical results not selected on basis of results
Other bias Other sources of bias	Low risk	None detected
Performance bias Blinding (participants and personnel)	Unclear risk	No information
Detection bias Blinding (outcome assessment)	Low risk	Appropriate method for assessing outcome. Ascertainment of outcome did not differ between groups – no information as to whether assessors were aware of intervention received. It is deemed unlikely that outcome was influenced by knowledge of the intervention received.
Attrition bias Incomplete outcome data	Unclear risk	No information given

Discussion

Surgical intervention remains the primary treatment for localised cutaneous melanoma, with wide local excision being the gold standard approach [21]. It is essential to ensure an adequate excision margin to achieve a favourable outcome while also minimising morbidity and allowing a cosmetically acceptable result. However, selecting the right excision margin can be complex. Guidelines from NICE and NCCN suggest that excision margins can be tailored based on the anatomical location and functional considerations, even though the evidence regarding the safety of this practice is limited [3,4]. This systematic review and meta-analysis is the first to directly examine the outcomes of 1 cm versus 2 cm excision margins for intermediate and thick cutaneous melanomas. 

In total, seven studies met the criteria for inclusion in this systematic review and meta-analysis (Figure 1). Of these, five studies looked at overall and locoregional recurrence comparing 1 cm or 2 cm margins (Figures 2, 3). Additionally, four studies evaluated the rate of distant recurrence in these two groups (Figure 4). There were no statistically significant differences observed in outcomes concerning excision margins overall for locoregional, or distant recurrence of melanomas when using 1 cm or 2 cm. This suggests that a narrower margin of 1 cm could be a safe approach for managing intermediate and thick melanomas, which is clinically significant since reducing the excision margin may limit tissue removal and associated morbidity without compromising oncological outcomes.

A study done by Utjés et al. [10] showed that using a 4 cm wide local excision margin versus a 2 cm excision margin did not affect melanoma-specific survival with an adjusted hazard ratio of 0.99 (95% CI 0.81 -1.20), p = 0.89. Similarly the results of this review indicate that taking a 2 cm margin compared to 1 cm didn't influence disease-free survival. Three studies analysing disease-free survival rates for patients treated with 1 cm and 2 cm excision margins found no statistically significant differences between the two groups [5,9,18]. Hunger et al. [5] employed Kaplan-Meier methods and Cox regression analysis, revealing no significant difference in disease-free survival between the 1 cm and 2 cm groups. Koskivuo et al. [9] using Kaplan-Meier analysis and Haydu et al.'s [18] multivariate analysis further supported it. The consistent findings across multiple statistical methods strengthen the evidence that wider excision margins (2 cm) may not provide additional benefit in terms of disease-free survival compared to narrower margins (1 cm). This further reinforces that a 1 cm margin may be a safe and effective approach in the surgical management of cutaneous melanomas, ultimately resulting in lower surgical morbidity while maintaining oncological safety. It is essential to note, however, that variability in follow-up periods and study designs could introduce bias. The data also indicates that the 1 cm excision margin leads to higher rates of direct primary closure with fewer patients requiring complex reconstructions, such as skin grafts or flaps, compared to 2 cm margins. This narrower margin benefits patients by allowing for quicker recovery times, reduced morbidity, lower costs and improved cosmetic outcomes [5,8,19,20].

Nonetheless, when comparing 1 cm and 2 cm excision margin groups, it is important to consider factors like tumour characteristics and the anatomical location of the melanomas. Narrower excision margins are often preferred particularly for melanomas on the face, trunk, and extremities, as they help preserve function and aesthetics. However, Doepker et al. [11] highlights the fact that head and neck melanomas carry a higher risk of recurrence. Additionally, the proximity of these melanomas to critical structures such as the eyes, nose, and mouth forces surgeons to find a balance between the appropriate excision margin and the functional and cosmetic needs of the patient [5,22]. This balance is highlighted by Rawlani et al. [19], who showed that reduced excision margins near critical facial structures like the eyelids and nose did not significantly increase local recurrence rates for melanomas ranging from 1.01-2.0 mm thick compared to standard margins. These findings suggest that narrower excision margins can effectively preserve function and appearance, particularly in anatomically sensitive regions, without compromising local control of the melanoma. However, careful decision-making is required, as margin size must still ensure the complete removal of the tumour while minimising the risk of recurrence.

It is important to recognise the significant barriers faced by patients from rural communities due to a limited access to plastic surgeons. Particularly in many U.S. states, rural areas lack active plastic surgeons, hindering access to both plastic and complex reconstructive care. These limitations can negatively impact clinical outcomes, particularly in patients with skin cancer such as melanoma, where insufficient plastic surgery resources and inadequate follow-up may contribute to the increased recurrence rates of melanoma. As a result, the selection of excision margin can often be influenced not only by the oncological properties but also by the availability to a plastic surgeon, making the margin decision shaped by local resource constraints [23].

This systematic review and meta-analysis supports narrower excision margins for managing intermediate and thick cutaneous melanomas, but several limitations must be noted. Only one randomised controlled trial and six observational studies met the inclusion criteria, limiting the generalisability of the findings. The lack of randomisation in Rawlani et al.'s [19] study introduced selection bias, while in the study by Hunger et al. [5], excision margin decisions depended on the consultant to whom patients were referred. The quality assessment of the observational studies using the Newcastle-Ottawa Scale revealed significant weaknesses, with five studies scoring poorly and only one achieving a fair outcome (Table 3). Although the randomised controlled trial had a low risk of bias, unclear allocation concealment and blinding raise concerns about the evidence's applicability to the general population (Table 4). According to NICE, patient follow-up should be offered for five years to patients with intermediate and high-risk melanomas [4]. However, some studies fail to meet this standard (Table 1). Therefore, to enhance validity of the results, more robust and consistent follow-up protocols should be implemented. Despite these limitations, this systematic review and meta-analysis provides a robust analysis of a large patient cohort, with a total of 4,613 patients included.

The authors of this systematic review and meta-analysis recommend conducting further research, including randomised controlled trials to strengthen the evidence base regarding excision margins of melanomas as most studies are retrospective cohort studies, which therefore introduce an inherent risk of bias. These studies should control for factors such as Breslow thickness as this is a critical determinant of disease outcome. Most studies meeting the inclusion criteria were retrospective cohort studies with discrepancies in follow-up length. Longer follow-up allows for more accurate understanding of the safety and efficacy of different excision margins, as well as providing more data to inform survival estimation models.

## Conclusions

The authors report the first systematic review and meta-analysis on the use of narrower excision margins in intermediate and thick cutaneous melanomas. The findings reveal no significant difference seen in the local, regional and distant recurrence when using 1 cm excision margin versus 2 cm margins. Furthermore, disease-free survival was comparable between the two groups. However, the strength of these conclusions is limited by the retrospective, non-randomised nature of most of the included studies, along with the associated risk of bias. To address these limitations, the authors advocate for further randomised controlled trials to provide further robust evidence. Despite these limitations, the authors believe that this study serves as a foundation for guiding the choice of narrower excision margins for melanoma.
